# “Your Digital Doctor Will Now See You”: A Narrative Review of VR and AI Technology in Chronic Illness Management

**DOI:** 10.3390/healthcare14020143

**Published:** 2026-01-06

**Authors:** Albert Łukasik, Milena Celebudzka, Arkadiusz Gut

**Affiliations:** 1 Department of Cognitive Science, Doctoral School of Social Sciences, Nicolaus Copernicus University, 87-100 Toruń, Poland; milena.celebudzka@gmail.com; 2 Department of Cognitive Science, Faculty of Philosophy and Social Sciences, Nicolaus Copernicus University, 87-100 Toruń, Poland; arkadiusz.gut@umk.pl

**Keywords:** virtual reality, artificial intelligence, digital therapy, chronic illness, patient engagement

## Abstract

**Highlights:**

**What are the main findings?**

- Immersive VR/MR and AI-driven virtual agents can significantly enhance engagement, motivation, and emotional well-being in patients with chronic illnesses by providing adaptive, personalized, and interactive therapeutic experiences.
- Despite their promise, these technologies face notable challenges, including technical limitations (e.g., latency, system dependence), ethical concerns (e.g., data privacy, algorithmic bias), and psychosocial risks (e.g., emotional over-attachment or discomfort from overly human-like avatars).

**What are the implications of the main findings?**

- Integrating VR/MR and AI into a unified tool for chronic illness management requires patient-centered design, clinician oversight, and transparent governance frameworks to ensure safety, empathy, and accessibility.
- When implemented responsibly, these technologies can complement traditional therapy by reducing treatment burden, supporting self-management, and improving long-term adherence and quality of life for patients with chronic conditions.

**Abstract:**

This narrative review examines how immersive virtual and mixed-reality (VR/MR) technologies, combined with AI-driven virtual agents, can support the prevention and long-term management of chronic illness. Chronic diseases represent a significant global health burden, and conventional care models often struggle to sustain patient engagement, motivation, and adherence over time. To address this gap, we conducted a narrative review of reviews and meta-analyses. We selected empirical studies published between 2020 and 2025, identified through searches in PubMed, Web of Science, and Google Scholar. The aim was to capture the state of the art in the integrated use of VR/MR and AI in chronic illness care, and to identify key opportunities, challenges, and considerations relevant to clinical practice. The reviewed evidence indicates that VR/MR interventions consistently enhance engagement, motivation, symptom coping, and emotional well-being, particularly in rehabilitation, pain management, and psychoeducation. At the same time, AI-driven conversational agents and virtual therapists add adaptive feedback, personalization, real-time monitoring, and continuity of care between clinical visits. However, persistent challenges are also reported, including technical limitations such as latency and system dependence, ethical concerns related to data privacy and algorithmic bias, as well as psychosocial risks such as emotional overattachment or discomfort arising from avatar design. Overall, the findings suggest that the most significant clinical value emerges when VR/MR and AI are deployed together rather than in isolation. When implemented with patient-centered design, clinician oversight, and transparent governance, these technologies can meaningfully support more engaging, personalized, and sustainable chronic illness management.

## 1. Introduction

### 1.1. The Current State of Chronic Illness Managment

Chronic illnesses are typically defined as health conditions that persist for one year or longer and require ongoing medical attention and/or limit activities of daily living [1]. These conditions, most commonly cardiovascular disease, diabetes, chronic respiratory disease, cancer, and multimorbidity clusters-account for substantial global morbidity, mortality, and healthcare expenditure, and their determinants span behavioral, psychosocial, health-system, and environmental domains [2]. Public health ethics frameworks remind us that the chronic disease agenda intersects with questions of resource allocation, prevention, equity, and the social determinants of health [3].

Chronic illness is commonly used as an umbrella term encompassing noncommunicable diseases with a prolonged time course, uncertain prognosis, and the need for sustained self-management [4]. Categorization is typically (1) by organ system or etiology (e.g., cardiometabolic, respiratory, oncologic, neurodegenerative); (2) by prevention stage: primary (risk-factor reduction before disease onset), secondary (screening/early detection), and tertiary (limiting progression and complications); and (3) by complexity, notably the presence of multimorbidity, where multiple conditions co-occur and interact across the life course to reshape treatment goals and capacities [5].

Living with chronic illness includes a treatment workload, the cumulative, dynamic, and multidimensional “burden of treatment” (e.g., learning, monitoring, attending appointments, adhering to complex regimens), which is distinct from disease burden and can itself cause non-adherence, poorer quality of life, and inefficiencies [6].

Not only that, effective care hinges on sustained self-management, a collaborative process that spans symptom control, behavior change, and psychosocial adaptation; self-management is influenced by personal/lifestyle factors, comorbidity, resources, environment, and healthcare system features, which can function as facilitators or barriers [4].

Adherence to agreed pharmacologic and lifestyle recommendations remains suboptimal, as some theoretically consistent effects on intentions and adherence show small-to-medium effects, underscoring both the promise and the limits of traditional social-cognitive approaches [7]. Also, persistent inequities in exposure, access, and outcomes demand culturally and contextually attuned interventions that span community and system levels [2]. Additionally, lifestyle change remains foundational. As some research suggests, among people with prediabetes, specific intervention programs demonstrated durable risk reduction in incident diabetes over 15 years for both intensive lifestyle intervention (−27%) and metformin (−18%) [8]. However, neither strategy reduced major cardiovascular events over 21 years [9]. In people with established type 2 diabetes, intensive weight loss may result in long-term weight support and improvements in intermediate risk factors but may not necessarily reduce the primary composite of cardiovascular events [10]. The contrast between strong effects on disease rates and intermediate markers, and attenuated or null effects on hard outcomes in specific populations, demonstrates the importance of population, timing, and intensity when judging lifestyle efficacy.

### 1.2. Therapy and Rehabilitation Become Virtual

Behavior change interventions for self-management are complex, multilevel, and benefit from systematic development using contemporary frameworks and reporting standards [11]. Meta-analytic work on adherence suggests that targeting attitudes, norms, and perceived behavioral control can improve intentions and behaviors. Still, effects are modest, implying a need to complement personalized techniques with environmental and service-delivery changes [7]. Self-management programs that account for treatment burden and the time demands of illnesses may enhance uptake and sustainability [5,6].

Against this backdrop, new technologies such as virtual or mixed reality (VR/MR) and artificial intelligence (AI) offer tools to potentially:

- reduce treatment burden, for example, by providing immersive symptom self-management training to lower pain perception during medical procedures [12] and enabling remote rehabilitation, such as for post-stroke patients [13,14],
- personalize behavior-change support by promoting health-oriented among individuals with chronic illness [15] or by changing the perception of the diagnosis [16],
- optimize clinical decision-making based on AI-driven insights [17,18] and resource allocation to prioritize the order of clinical actions, improving workflow efficiency [19].

These solutions should be judged against the comparative benchmarks outlined above: effects on patient-important outcomes, demonstrable burden reduction, and equity in access and impact [2,3]. Immersive VR interventions increasingly complement traditional rehabilitation and psychotherapy by providing embodied, experiential learning environments that enhance patient engagement and adherence [12]. Yet, their clinical use requires deliberate preparation and oversight, such as gradually introducing the patient to the virtual environment, ensuring comfort with the headset, confirming visibility and safety, and establishing clear behavioral cues, for example, agreed-upon gestures for pausing or exiting the session [13]. For many patients, especially children, gamified VR experiences that integrate movement and feedback can transform rehabilitation from a passive to an interactive process [14]. Such experiences can facilitate perceptual learning, relieve chronic pain through distraction and immersion, and promote emotional resilience. However, these benefits are counterbalanced by limitations, such as initial discomfort, fear, or overreliance on novelty [15].

Similar to VR and MR technology, AI-based systems, including chatbots, embodied virtual therapists, and adaptive dialog agents, also offer scalable and personalized extensions of traditional care models [16]. From a technological perspective, these systems can support patients between therapy sessions by reminding them of exercises, tracking physiological signals from wearables, and adjusting interventions in real time. They may also enhance the VR/MR experience by optimizing interactivity and individual adaptation. Yet these solutions raise practical and ethical challenges: dependence on servers and latency issues, constraints on local large-language-model (LLM) deployment, and the risk of inaccurate or hallucinatory outputs [19]. Designing AI “therapists” thus requires careful tuning to balance responsiveness with safety, privacy protection, and transparency regarding access to medical or biometric data. From a social and emotional standpoint, additional factors emerge—the avoidance of uncanny valley effects, the selection of appropriate voices and communicative styles, and the potential risks of over-personalization or emotional attachment, particularly among patients with concurrent mental health vulnerabilities [17,18,20].

In the following paper, we address both opportunities and challenges associated with the use of the latest technology in the field of chronic illness. In the first part of this review, we explore the role of virtual and mixed reality in the prevention and management of chronic illness. The second part of this review addresses the current and future use of AI in virtual rehabilitation, along with its pros and cons, including both technological and psycho-social challenges. Our goal is to outline the current state-of-the-art of VR/AI-based approaches to treat or prevent chronic illness, identify limitations at the technical and patient-oriented levels, and provide initial guidelines for applying such technology in clinical care.

## 2. Methods

This article was conducted as a narrative review with search procedures focused mainly on reviews and meta-analyses. The review aimed to present the current state of knowledge, identify emerging trends and research gaps, and provide a synthetic, interdisciplinary integration of findings on the use of virtual and mixed reality and artificial intelligence in chronic illness management. Rather than performing a quantitative synthesis, this approach was chosen to capture the rapidly evolving and heterogeneous evidence base at the intersection of immersive technologies, AI-driven virtual agents (including autonomous avatars), and chatbots, and to support a conceptually oriented, clinically relevant discussion.

A literature search was conducted between 2020 and 2025, as this timeline enabled us to identify state-of-the-art VR and AI solutions in clinical care. Three search engines were used: Google Scholar, PubMed, and Web of Science. The following keywords combinations were applied: “chatbot” + “chronic illness” + “review”, “chatbot” + “chronic illness” + “meta-analysis”, “mixed reality” + “chronic illness” + “review”, “mixed reality” + “chronic illness” + “meta-analysis”, “virtual reality” + “chronic illness” + “review”, “virtual reality” + “chronic illness” + “meta-analysis”. 

The following results were obtained for the keyword combinations above:

- “chatbot” + “chronic illness” + “review” (Google Scholar, n = 12,100; Pubmed, n = 33; Web of Science, n = 78),
- “chatbot” + “chronic illness” + “meta-analysis” (Google Scholar, n = 17,000; Pubmed, n = 1; Web of Science, n = 85),
- “mixed reality” + “chronic illness” + “review” (Google Scholar, n = 16,800; Pubmed, n = 21; Web of Science, n = 5),
- “mixed reality” + “chronic illness” + “meta-analysis” (Google Scholar, n = 17,000; Pubmed, n = 1; Web of Science, n = 2),
- “virtual reality” + “chronic illness” + “review” (Google Scholar, n = 18,000; Pubmed, n = 82; Web of Science, n = 54),
- “virtual reality” + “chronic illness” + “meta-analysis” (Google Scholar, n = 16,900; Pubmed, n = 24; Web of Science, n = 54).

**Figure 1:**
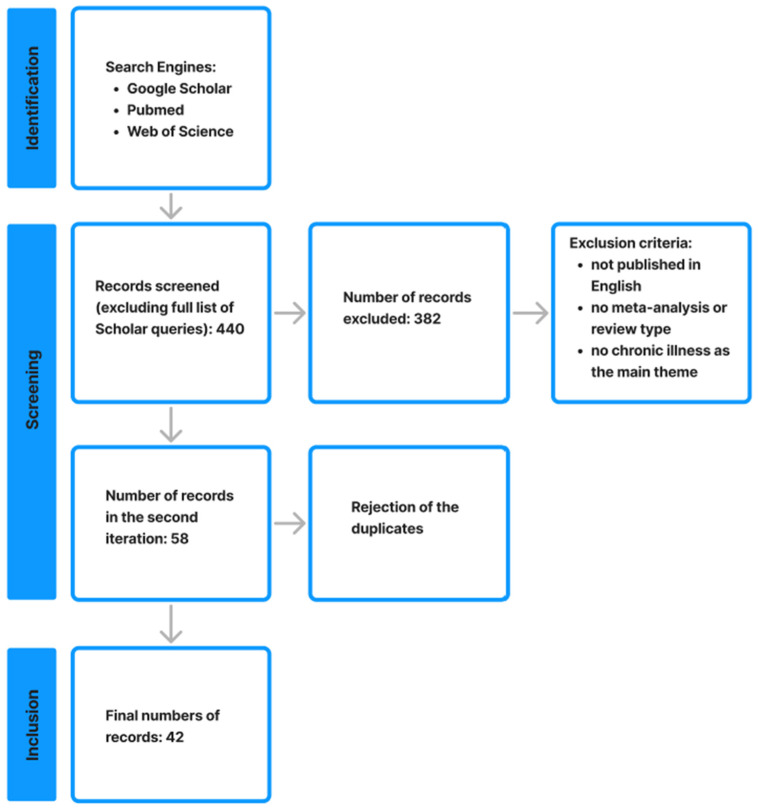
Schematic presentation of the literature review with inclusion and exclusion criteria.

Since Google Scholar returned a large number of results for each search, we focused only on the first 10 queries that yielded articles relevant to our review. Articles were excluded if they were not published in English, were not directly related to chronic illness management, or were duplicates. Reviews addressing VR or AI in healthcare without a chronic illness focus were also excluded. Following screening, a total of 42 review and meta-analytic papers were included in the final synthesis. The results are presented narratively, with emphasis on thematic patterns, technological and clinical trends, conceptual gaps, and practical implications for clinicians rather than exhaustive enumeration of individual studies. Figure 1 demonstrates the flowchart of the literature review procedure.

## 3. Augmented, Virtual, and Mixed Reality: VR in Healthcare

### 3.1. Practical Implementation of VR for Maintaining Quality of Life

Thanks to technological advances, non-invasive XR (Extended Reality) methods are increasingly used in education, therapy, and rehabilitation of chronic diseases. XR is a collective term encompassing all technologies of AR, VR, and mixed reality (MR) [21]. AR involves overlaying digital elements onto the real world. VR is a technology that completely transports the user into a virtual, computer-generated, and isolated environment. MR, in turn, is a more advanced form that combines elements of virtual and augmented reality. In MR, digital and physical objects coexist and can interact in real time [22].

VR/MR systems provide users with multisensory experiences that, depending on the degree of immersion, can range from non-immersive to fully immersive [22,23]. Immersive systems use head-mounted devices (VR headsets) that display a 360-degree environment, allowing users to experience a greater sense of presence [23]. The importance of immersion in VR/MR technology is significant, as it can enhance engagement and satisfaction with the VR experience [24]. Properly designed VR environments with high levels of immersion can therefore contribute to the effectiveness of interventions (e.g., rehabilitation exercises, psychological therapy, or relaxation) [25,26,27,28].

The use of VR/MR in the therapy of chronic diseases opens up a wide range of therapeutic possibilities for adult, pediatric, and elderly patients alike [29,30,31,32]. These technologies enable cognitive function training, attention exercises, relaxation, and motor rehabilitation, both for fine and gross motor skills [33,34,35,36,37]. Due to the immersive nature of VR, patients are more willing to participate in the therapeutic process, as tasks performed in a virtual environment are often more engaging, enjoyable, and less stressful than those in traditional methods [38,39]. This is particularly evident in the case of children, for whom elements of gamification and visually appealing design are essential for maintaining motivation and concentration [40]. In VR, various scenarios can be recreated and tailored to the patient’s needs, for example, motor exercises in a forest setting, educational games teaching about one’s illness, or simulations of stressful situations to help patients become accustomed to them [41,42,43].

Importantly, VR/MR should not be regarded as a competing method to traditional therapy, but rather as its complement [44]. Contemporary research clearly indicates that VR can serve as a supportive tool that enhances the effectiveness of therapy, for example, in phobia and anxiety treatment in comparison to traditional therapy sessions with no VR involvement [31]. This is particularly relevant in the context of long-term therapies, as chronic diseases require continuous effort and sustained patient motivation. Regular immersive experiences can help maintain engagement and facilitate the therapeutic process [45,46,47,48].

The possibilities of using VR/MR in the therapy of chronic diseases are vast. In the case of physical conditions like chronic back pain, fibromyalgia, or osteoarthritis, VR supports movement-based treatment and helps reduce fear of movement (kinesiophobia) [49,50]. Interactive, controlled scenarios engage the patient in rebuilding physical ability and self-confidence. In neurological disorders (e.g., multiple sclerosis, stroke, Parkinson’s disease) [51], VR is used to train balance and motor coordination and to stimulate neuroplasticity [52,53]. For mental health disorders such as depression, anxiety, or PTSD, VR provides the opportunity for controlled exposure to stimuli and relaxation in a safe environment [54].

This technology is also applied in the therapy of cancer, respiratory, metabolic, and autoimmune diseases [55,56,57]. Among adult patients, including seniors, those undergoing oncological or rheumatological treatment use VR to reduce pain and anxiety, support gentle physical activation, conduct cognitive training, and participate in psychoeducation that helps them understand the complexity of their disease and learn coping strategies [58,59]. For patients with COPD or asthma, VR technology supports breathing exercises and improves respiratory control [39,42]. In metabolic diseases, it serves as an educational tool that enhances understanding of treatment mechanisms and self-management. Particularly significant benefits of VR/MR interventions have been observed in individuals with obesity, both adults and children. Interactive motion-based VR games combine physical exercise with entertainment [46]. Additionally, VR is used for training healthy eating habits and to monitor diet [43]. The observed effects of such intervention are improved self-control, increased physical activity, and enhanced body satisfaction and health-related self-efficacy [60,61].

VR is also used as a tool for emotional and cognitive support, especially among children and adolescents [62]. An example is the use of immersive educational games for children with diabetes, which teach them to interpret glucose readings, make therapeutic decisions, and administer insulin correctly [63]. As a result, children develop a sense of agency and gain a better understanding of their illness, which positively influences their self-esteem and daily functioning. Similar mechanisms are also observed in adults, for whom immersive VR education helps adapt to a new diagnosis, improves understanding of their health condition, and supports emotional self-regulation [64].

The education of children and adolescents with chronic illnesses is another aspect being tackled with the help of VR [65]. Extended hospital stays, frequent absences from school, and intensive rehabilitation therapies significantly affect the educational process. VR enables the integration of learning elements with therapy by providing engaging educational content that meets children’s cognitive needs [46]. Studies have shown that VR supports the development of cognitive abilities, improving working memory, attention, and visuomotor coordination [33]. Through VR, children can, for example, learn geography by “exploring” the world in 360 degrees, develop language skills through dialog with avatars, or practice mathematics by solving problems in a 3D space.

### 3.2. Challenges in Implementing VR in Chronic Illness Prevention, Therapy, and Enhancing the Quality of Life

Immersive VR and MR technologies are becoming increasingly affordable and technically accessible. The cost of purchasing simple home-use sets is relatively low, allowing patients to continue rehabilitation or psychological exercises at home without frequent travel to therapy centers [52]. Such solutions reduce treatment costs while supporting patient independence. VR can also serve as an alternative for individuals who, due to economic, geographical, or mobility-related reasons, cannot regularly attend therapy in clinical facilities [27].

An additional advantage of VR is the possibility of complete personalization of experiences. The user can adjust the environment, the level of task difficulty, and the mode of interaction (e.g., controllers, hand movement, eye tracking), which enhances comfort and the effectiveness of the intervention [22]. For individuals with limited mobility, VR can serve as the only window to the world, allowing them to “walk” through a forest, admire mountains, or be surrounded by wildlife. Research indicates that contact with nature, even in simulated form, can still reduce stress levels and support recovery [28].

In addition to regular use, the effectiveness of VR/MR therapy also depends on the patient’s age and technological competence [39]. Among children and adolescents, there is a high level of acceptance of this form of therapy. It is perceived as attractive and modern [58]. Adults also often engage with VR with interest, particularly for relaxation and cognitive therapies [41]. For older adults, prior training and therapist support may be necessary, as barriers can include technophobia or a lack of previous experience with digital tools [31,32].

Despite its numerous benefits, the use of VR/MR in therapy also poses several challenges. The average duration of a single therapeutic VR session is typically around 15–20 min, as indicated by most systematic reviews; however, there are no clear guidelines regarding the optimal session length [66]. 

Several clinical trials suggest that this duration balances therapeutic engagement with user tolerance, especially in populations with reduced stamina or cognitive load capacity [66]. Studies also report that prolonging VR sessions beyond 30 min may increase the risk of mental fatigue or reduced effectiveness due to habituation [66,67]. For this reason, some researchers advocate for embedding self-regulation tools within applications, such as built-in timers, adaptive task pacing, and session-ending reminders [68].

Longer exposures may lead to side effects (simulator sickness), such as dizziness, nausea, vomiting, or eye strain, which is particularly present among individuals with sensory sensitivity or older adults [69]. These adverse reactions are often linked to sensory conflict between visual and vestibular input, low frame rates, or poor calibration of the virtual environment [70]. Studies indicate that the likelihood and intensity of these symptoms increase significantly after continuous exposure exceeding 30–40 min, especially without breaks or adaptation periods [69]. To mitigate such effects, gradual familiarization, shorter initial sessions, and adjustable environmental parameters are recommended, particularly for first-time users and vulnerable groups [71].

Excessive or prolonged exposure and the advanced immersive features of VR/MR, like engaging interactivity, personalized content, and instant accessibility, may contribute to addictive behaviors [72,73]. At the same time, these very characteristics can be used therapeutically, illustrating the dual nature of VR effects. Addiction to XR technology or environments may manifest when a patient loses track of time, wishes to prolong sessions repeatedly, or neglects alternative and conventional therapeutic methods [72]. Therefore, in a therapeutic context, special caution is recommended, including regular evaluation of patient progress and screening for symptoms of addiction [74].

Additional important considerations include training personnel and caregivers and adapting the therapeutic space to the specific application, environment, or game [75]. Individuals with sensory sensitivities should be gradually introduced to immersive experiences [76]. It is essential to identify and implement a personalized environment tailored to each individual, allowing them to interact as accessibly as possible, not only through movement or navigation controllers but also via hand tracking or eye-gaze control [75].

Notably, the number of therapeutic applications that offer such personalization options remains limited. Few currently provide the ability to choose the mode of interaction (controllers, hands, eye tracking), automatically monitor progress, adjust task difficulty, and deliver real-time feedback [39].

A crucial ongoing challenge is designing VR/MR environments that adhere to usability (UX) principles. A straightforward interface, explicit instructions, sensory alignment, and intuitive navigation are essential for effective and safe use of VR in therapy, particularly among vulnerable groups such as children and older adults [40,46]. The complexity of immersive experiences necessitates close collaboration among psychologists, therapists, computer scientists, and designers. 

While VR technology can be suited to patient’s needs by providing immersive, safe and engaging space it also poses risks associated with the patient’s individual characteristic such as proness to headaches or nasuea caused be excessive movement of the camera inside the virtual world, risk of getting addicted to this type of intervention, and the amount of preliminary training that may be required to become familiar with the headset. Both researchers and medical staff should also be aware that some technological challenges depend on the type of headset, such as fidelity, field of view, or the headset’s weight.

There are now several VR applications on the market that leverage the potential of immersion for therapeutic purposes. Examples include applications developed by the company Unicorn VR World [77]. The first, “Strefa ciszy VR” (Silence Zone VR), is designed for users of all ages and offers four diverse environments that enable concentration training, relaxation, and mindfulness exercises.

The second, TheraplyVR, is a platform primarily aimed at therapy for younger children. It provides relaxation scenarios, mindfulness exercises, psychoeducation, and cognitive and executive function training (e.g., auditory perception, visual perception, visuomotor and audiomotor coordination), which can be performed both in the therapist’s office and independently at home.

The application transports the patient into a fairy-tale world guided by a character named “Niko.” The guide’s role is to instruct, assist, support, encourage, and praise the user by providing feedback. The app is fully controlled through a mobile application, allowing the therapist, parent, or caregiver to select the appropriate scenario for the child. Moreover, it includes features for recording and monitoring patient progress and adjusting the difficulty level.

## 4. AI-Influenced Revolution in VR Treatment Interventions

### 4.1. A Virtual Environment with a Virtual Therapist as a Way to Enhance Motivation to Complete the Session

Artificial intelligence has begun to fundamentally transform how virtual and augmented reality systems are conceived and deployed in healthcare, shifting them from static or preprogrammed environments toward adaptive, data-driven ecosystems. Traditional virtual systems based on virtual reality simulations provide valuable immersive experiences for pain management, rehabilitation, or education, but they rely mainly on scenarios or clinician-controlled parameters [78]. When enhanced with artificial intelligence, however, these technologies become responsive systems capable of learning from user data and adapting in real time to meet the patients’ needs by providing virtual agents [79]. Algorithms can analyze physiological, behavioral, or cognitive metrics, such as heart rate, movement patterns, or emotional cues, to dynamically adjust the virtual environment [80]. This capability allows clinicians to personalize the level of challenge, sensory stimulation, or feedback based on the patient’s progress, creating a closed-loop therapeutic experience that optimizes engagement and outcomes such as interpreting data from wearable devices [80]. In data-driven virtual rehabilitation platforms, for example, artificial intelligence continuously interprets sensor and motion-capture data to modify intensity or provide corrective guidance, effectively mimicking a human therapist’s adaptive responses [81]. Such integration turns virtual sessions from static supporting tools into intelligent systems of care that improve with each patient’s condition, enhancing motivation and long-term adherence to therapy programs [82].

A further evolution involves the emergence of AI-driven virtual therapists or conversational agents that accompany patients throughout immersive rehabilitation or cognitive therapy programs [83]. These systems, powered by natural language processing and affective computing, simulate empathetic human interaction, offering guidance, encouragement, and psychoeducational support during virtual sessions [84]. For instance, autonomous avatars (those controlled by large language models) can track progress in real time and modify their communication style or therapeutic content according to a patient’s performance and emotional state [49]. Within chronic pain management, hybrid AI-VR cognitive therapies employ intelligent agents that adjust tasks, reinforce coping strategies, and deliver personalized relaxation scenarios [85]. On a broader scale, the convergence of virtual reality, explainable artificial intelligence, and blockchain technologies within metaverse-based systems enables secure, trustworthy, and immersive remote healthcare experiences, in which patients and clinicians interact through avatars in shared virtual environments [86]. These environments not only extend access to specialized care but also offer continuous monitoring and feedback loops that support behavioral change and self-management. In essence, artificial intelligence augments virtual sessions by infusing adaptability, emotional intelligence, and interactivity into virtual healthcare, paving the way for deeply personalized and sustainable management of chronic illness. This solution, however, is not flawless and carries a few potential problems worth investigating.

### 4.2. Challenges in AI-Based Virtual Healthcare from a Technical and Social Perspective

AI-driven mobile agents or conversational assistants can monitor physiological or behavioral data gathered from wearable devices and prompt users to perform exercises, take medication, or log symptoms [80,87]. In extended-reality therapeutic environments, these agents can even adjust feedback or environmental cues in real time based on patient data, thereby maintaining engagement and therapeutic continuity [88,89]. Although AI-based systems can also serve as supporting therapeutic aids, ensuring continuity of care between clinical visits by issuing reminders, providing motivational feedback, and guiding patients through daily rehabilitation or self-management tasks, there are at least a few technological problems. One of them is reliance on cloud-based artificial intelligence architectures: when central servers or remote processing nodes fail or experience latency, therapy continuity may be disrupted, jeopardizing patient safety and undermining trust in the technology [90]. This dependency is particularly problematic in remote areas with limited resources, where connectivity is unstable, a point emphasized in both pediatric and adult chronic care contexts [91].

Emerging approaches propose localized large language models and edge artificial intelligence as partial solutions to this problem, enabling agents and adaptive virtual environments to function effectively from centralized infrastructure. By processing data locally on a headset, smartphone, or hospital server, artificial systems can continue to deliver reminders, evaluate progress, and guide the patient even during network interruptions [92]. Yet, local AI language models face constraints in computational capacity, energy demand, and data update frequency, making it challenging to achieve autonomy and real-time synchronization with broader medical systems. Moreover, decentralizing computation introduces new privacy and maintenance challenges, particularly regarding secure updates and data standardization across devices. Therefore, while localized artificial intelligence can mitigate some risks associated with central server outages, a hybrid model combining computing for critical real-time interactions with periodic cloud synchronization appears to be the most viable pathway toward a resilient AI-supported therapy ecosystem for chronic illness management.

The development of LLM-based virtual therapists for chronic illness management requires precise tuning to balance accuracy, safety, and responsiveness [93]. Unlike general-purpose chatbots trained on open-domain data, dedicated conversational agents must be constrained by a clinically validated database, such as disease-specific guidelines and behavioral change frameworks, to minimize the risk of hallucinations and misinformation [94]. Some studies have shown that LLM-driven diagnostic or counseling systems can achieve high accuracy when fine-tuned on domain-specific datasets, achieving around 97% diagnostic precision in chronic disease contexts [95], but can also produce unsafe or biased recommendations if overgeneralized [96]. To mitigate such risks, developers employ reinforcement learning from human feedback combined with medical rule-based constraints that restrict outputs to evidence-based options. This allows the virtual therapist to offer informative, contextually aware guidance without overstepping into unverified therapeutic territory. However, excessive filtering or restrictive prompting can render the agent unresponsive or “robotic,” undermining patient engagement and adherence, which are crucial for long-term behavioral change [97]. Narrowly speaking, fine-tuning should not only prioritize factual accuracy but also maintain a natural, empathetic communication flow that sustains patient motivation during self-management.

A key distinction between therapy-oriented chatbots and general conversational systems lies in their data provenance and functional purpose. While open-domain models like ChatGPT, Gemini, or Claude draw from vast, heterogeneous internet sources, medical or condition-specific chatbots rely on structured, peer-reviewed medical knowledge bases and real-world patient data integrated through clinical decision-support frameworks [98,99]. Therapeutic chatbots such as Wysa [100] are explicitly programmed to avoid prescriptive statements and instead deliver motivational interviewing, cognitive reframing, and adherence reminders. Conversely, general chatbots lack embedded clinical reasoning, which can increase the risk of generating speculative or harmful advice. Studies on generative AI systems like ERNIE Bot further reveal that, while these systems surpass average physician diagnostic accuracy, they also tend toward overprescription and uneven care delivery, underscoring the ethical and safety implications of insufficient model constraints [101]. Ultimately, safe deployment of virtual therapists requires hybrid architectures that merge domain-limited large language models, clinician oversight, and explainable AI methods to ensure the system remains both medically reliable and empathetically responsive, rather than an unregulated conversational proxy [102].

Another technical aspect to consider is the type of communication with the chatbot. The choice between text-based and voice-based communication in AI-powered healthcare systems carries significant technical and accessibility implications, especially for patients with chronic conditions who may face sensory, cognitive, or motor challenges [103]. Voice interfaces, as demonstrated in recent studies, can markedly enhance accessibility and engagement for older adults and patients with limited literacy or dexterity, enabling hands-free interaction through speech recognition systems [103,104]. However, voice input introduces latency and interpretation challenges, as speech-to-text algorithms are highly susceptible to background noise and language diversity, which can result in medical terminology or produce inaccurate responses [103,105]. This is particularly problematic in multilingual or rural settings, where dialectal variation may exceed the scope of training for deep neural network speech models [106]. Conversely, text-based chatbots offer higher precision and auditability, allowing users and clinicians to verify conversation history, yet they exclude individuals with visual or motor impairments [103].

Latency is another critical factor distinguishing the two modalities: while text exchanges are primarily constrained by network response time, voice systems require dual processing speech recognition and semantic interpretation, often resulting in increased end-to-end delay [107]. These delays can compromise the sensational flow of the interaction and, over time, reduce user trust and engagement in interactions such as rehabilitation coaching. Hybrid solutions that integrate both voice and text, allowing seamless switching between voice and text, have been shown to enhance usability and inclusivity, particularly when implemented with multilingual support and adaptive learning algorithms that refine recognition accuracy based on individual users’ speech patterns [108,109]. Nevertheless, the technical trade-off between accessibility, latency, and interpretive accuracy remains a key challenge in deploying voice-enabled tools for chronic illness management, pinpointing the need for localized language models and real-time error correction mechanisms to ensure both responsiveness and clinical reliability.

Last, and perhaps most important, technical aspect of implementing AI-integrated virtual systems in managing chronic illness is data privacy. As these systems gain access to sensitive medical records and continuous biometric data from wearables, they are beginning to raise profound data privacy, ethical, and regulatory challenges. AI-driven platforms aggregate multimodal datasets, electronic health records, genomic data, sensor streams, and conversational logs in order to enable predictive and personalized care. Still, such a system amplifies the risk of unauthorized disclosure and misuse [110]. While predictive analytics and natural language models can enhance individualized treatment and early intervention, they depend heavily on large-scale, personally identifiable datasets, making patient consent, data minimization, and secure storage critical to prevent privacy breaches [110]. The ethical concern is exacerbated when LLM-based systems retain conversational memory or use external API calls for inference mechanisms that expose protected health information to third-party servers, violating confidentiality standards such as GDPR [97]. Moreover, artificial systems’ ability to infer emotional or behavioral states from voice tone, facial data, or physiological signals collected through wearable sensors introduces new layers of biometric surveillance and potential stigmatization if such insights are misinterpreted or shared beyond the care context [88].

To address these risks, some scholars call for explainable artificial intelligence frameworks, federated learning architectures, and localized large language model deployment that allow computation on dedicated devices or within secure hospital networks, minimizing data exposure while maintaining model accuracy [111]. However, implementing these solutions requires balancing transparency with technical feasibility. While local models enhance privacy, they may lack the computational power and continual updates of cloud-based systems, leading to disparities in care quality. Ethical AI-based systems demand robust governance, involving algorithmic auditing, patient education, and dynamic consent mechanisms that allow individuals to control the scope of their data use [112]. Ultimately, safeguarding patient autonomy and data integrity must remain central to AI-driven healthcare innovation; without robust trust frameworks, even the most advanced LLM-powered systems risk undermining the very patient-centered care they aim to deliver.

Besides technical challenges, integrating virtual solutions in healthcare with AI-based approaches creates additional areas to explore regarding the social and emotional consequences of patient-artificial agent interactions. One of those challenges is associated with well-known phenomena, such as the uncanny valley—the discomfort elicited when artificial agents appear or behave almost, but not quite, human [113]. As this effect is essential to ensuring trust and emotional comfort [114], the human tendency to attribute mental states to artificial agents depends on how closely the agent’s social cues align with genuine human expectations. When avatars demonstrate near-human but slightly incongruent facial movements, speech timing, or emotional tone, users may experience unease that interrupts therapeutic engagement. This is particularly relevant in chronic care contexts, where sustained emotional connection with a virtual therapist is critical to adherence and self-management. Emotionally adaptive avatars, whose expressions and vocal prosody dynamically match users’ affect, can enhance perceived empathy and social presence, thereby mitigating the uncanny effect [114]. In contrast, avatars with overly photorealistic or monotone speech patterns risk eliciting cognitive dissonance or emotional detachment. One of the potential solutions is a cartoon-like avatar that would elicit warm emotions from the patient’s perspective.

As previously stated, beyond visual realism, voice and linguistic expression contribute strongly to the uncanny valley in healthcare interactions. Studies on AI-based virtual human systems such as BeCalm [115] show that participants perceive virtual agents as more trustworthy and “warm” when their tone and conversational rhythm are natural but not overly human-like. Similarly, voice assistants supporting patients with noncommunicable diseases are well accepted when their voices balance clarity with emotional neutrality, avoiding exaggerated inflections that mimic human empathy [116]. The same principle extends to text-based interactions, where excessive personalization or anthropomorphic phrasing can feel manipulative or insincere. Designing effective VR-AI therapeutic agents thus requires calibrating the realism of the avatar’s face, voice, and dialog style to remain within a “comfort zone” of familiarity, human enough to foster empathy, but artificial enough to avoid the cognitive dissonance of near-human imitation. This balance is foundational to sustaining emotional engagement, reducing anxiety, and fostering the trust needed for long-term virtual therapy adherence. One of the possible solutions is to implement virtual characters that already have a scripted human voice recording. Although the character cannot respond to users’ feedback in real-time, this solution can be great for “humanizing” virtual therapists (by using a real voice) in selected scenarios that do not require response adaptability.

Another characteristic of virtual avatars, especially those integrated with the LLM-based communication, is the personalization of both their behavior and appearance. While personalization is a cornerstone of user engagement in immersive healthcare technologies, granting patients extensive control over a virtual therapist’s speech, appearance, and behavioral style introduces both opportunities and psychological risks. On the one hand, allowing users to tailor their virtual environment and therapeutic agents can enhance motivation, immersion, and perceived agency, which are critical components for long-term adherence in chronic illness management [117]. AI-driven personalization of dialog and tone can increase empathy and engagement, as evidenced by AI-based educational systems for chronic pain management that dynamically adapt to individual cognitive and emotional needs [118]. However, excessive customization, particularly of avatar anthropomorphism, voice warmth, or affective responsiveness, risks emotional overattachment or therapeutic displacement, where pain focuses more on tailoring the system than on progress in therapy [119,120].

From a psychosocial perspective, over-personalization blurs the line between therapeutic empathy and artificial companionship, potentially reinforcing avoidance behaviors or unrealistic expectations of human therapists. While patient agency in virtual design supports empowerment, ethical safeguards are needed to prevent “too emotional” interaction with avatars or maladaptive parasocial bonds [121]. Furthermore, personalization of virtual therapists’ voices or personalities may inadvertently create dependency, reducing patient autonomy and emotional resilience, especially in chronic disease contexts where long-term engagement is required [122]. The optimal design thus involves guided personalization, in which patients can adjust superficial aspects, such as environment, tone, or gender, but not. Still, scripts and behavioral parameters remain clinically anchored and validated by psychological expertise [123]. Clinicians who are able to apply such AI-powered solutions should clearly inform patients about the possibilities and limitations of virtual therapists, while maintaining AI explainability. Calibrated balance preserves engagement and comfort while minimizing emotional entanglement, ensuring that the AI-driven therapeutic process remains both ethically responsible and effective in supporting clinicians’ work. Introducing AI to patients should therefore focus on both opportunities associated with its digital form, like large training datasets, availability or constant monitoring, but should also highlight the potential drawbacks in the form of social problems (emotional attachment) and technical challenges (hallucinations).

It is crucial to recognize that many chronic conditions are accompanied by mental health comorbidities, including depression, anxiety, or psychotic features [122], which heighten emotional vulnerability to artificial agents. Patients coping with chronic illness often experience isolation and cognitive fatigue, which may predispose them to forming parasocial or emotionally dependent relationships with AI-driven virtual therapists [121]. The “AI companion” literature demonstrates that emotional trust and interactive engagement can simulate intimacy and attachment, fostering a perceived sense of connection that may be particularly attractive to those with diminished social support [82,123]. However, this exact mechanism carries psychological risk: emotionally fragile users may develop excessive attachment, dependency, or even delusional interpretations of AI interactions, as observed in emerging cases of “AI-induced psychosis” [124]. Individuals with psychotic vulnerability may misinterpret the model’s memory functions or anthropomorphic cues as signs of sentience, reinforcing delusions of reference or control. Moreover, biases embedded in training data can unintentionally perpetuate stereotypes related to gender, race, or mental illness, subtly shaping the user’s self-concept and exacerbating internalized stigma [125]. This creates the need for therapeutic safeguards like emotional boundary protocols, clinician oversight, and transparency about artificial intelligence limitations to prevent emotional overidentification and epistemic instability in psychologically vulnerable users. Ultimately, while AI-mediated care offers accessibility and support, ethical design must ensure that such systems remain psychologically safe, particularly for patients whose mental health challenges make them more susceptible to digital emotional influence.

## 5. Safe Implementation of AI/VR Technology in Tackling Chronic Illness

To ensure safe deployment of AI- and VR-based therapies, concrete operational safeguards must accompany technological implementation in clinical settings. Empirical studies and recent systematic reviews consistently recommend established session durations (around 15–30 min per VR exposure), with mandatory breaks to reduce cybersickness, emotional overload, and cognitive fatigue, particularly in patients with anxiety and depression [94]. Contraindications for VR-based interventions should be formally screened and documented, including a history of photosensitive epilepsy, severe vestibular disorders, active psychosis, dissociative disorders, or high suicide risk, as emphasized in clinical AI and pediatric chronic-care reviews [106]. From a system-design perspective, emerging clinical risk frameworks advocate a layered safety model: (1) scripted therapeutic boundaries, whereby virtual agents are restricted to evidence-based psychoeducation, motivational interviewing, or CBT-derived content and explicitly prevented from diagnostic or prescriptive claims [100]; (2) automated alert thresholds, in which physiological signals (e.g., sustained heart-rate elevation), behavioral disengagement, or linguistic markers of distress trigger session interruption and clinician escalation [94]; and (3) explainable AI protocols, enabling clinicians and patients to understand the rationale behind system adaptations, thereby supporting transparent, informed consent, and trust [88]. Across recent randomized trials, scoping reviews, and meta-analyses, there is a strong consensus that hybrid human–AI oversight, rather than fully autonomous deployment, is essential to mitigate risks such as emotional overattachment, misinformation, or inappropriate therapeutic authority. Embedding these safeguards aligns AI-enhanced VR therapy with patient-centered care principles and emerging regulatory expectations, ensuring that adaptive virtual interventions for chronic illness remain clinically safe, ethically responsible, and therapeutically effective.

## 6. Discussion

This narrative review examined how immersive VR/MR and AI-driven conversational/virtual agents can complement standard approaches to chronic illness prevention and long-term management, emphasizing the patient’s perspective while acknowledging implementation issues that matter to clinicians and health systems. Across the literature, three cross-cutting themes emerge:

- promise for short-term symptom relief, engagement, and self-management support,
- uneven evidence for durable clinical benefit and real-world deployment,
- design, ethical, and workflow requirements that determine whether these technologies reduce, rather than add to, the burden of living with chronic disease.

Engagement and short-term outcomes are consistently positive. CAs and chatbots supporting mental health and self-management generally show small-to-moderate short-term benefits across depressive and anxiety symptoms, distress, well-being, and condition-specific complaints; effects tend to be larger when systems personalize content and respond empathically, and with longer interaction time. Reviews focused on randomized trials also find good feasibility and acceptability, with completion rates near 80% and encouraging effects on lifestyle change and psychosocial outcomes, albeit with heterogeneity and variable risk of bias. In chronic conditions specifically, users report chatbots that are helpful for self-monitoring and behavior change, though technical reporting and efficacy evidence remain uneven. Scoping work in mental health echoes these patterns, promising symptom improvement and behavior change, with usability/integration challenges still limiting.

Immersive VR can make rehabilitation, psychoeducation, and exposure feel doable and even enjoyable. For many patients, especially children and adolescents, gamified and embodied VR experiences increase motivation, reduce procedural anxiety, and enable graded exposure or skills rehearsal in safe environments. Virtual agents that adapt nonverbally in real time can further improve perceived rapport and session effectiveness, which is an insight directly relevant to sustaining adherence in chronic care.

Pediatrics and multimorbidity highlight both opportunity and risk. For children and adolescents with chronic illness, AI systems span monitoring, prediction, and coaching, but most studies are small or retrospective. With that in mind, prospective, real-world evaluations are still needed, along with attention to consent, culture, and stakeholder attitudes.

Prevention and targets associated with life seem achievable in the near term. Where disease modification is slow or uncertain, these tools can still lighten the “treatment workload” by providing on-demand coaching, symptom tracking, and immediate prompts between visits, contributing to better daily functioning and self-efficacy even if “hard” outcomes take longer to shift.

Durability and generalizability remain limited. Meta-analytic gains often attenuate at longer follow-up, and many studies use convenience samples or single-condition contexts; reporting of technical architecture and mechanisms is frequently sparse, constraining replication and transfer to diverse chronic populations.

According to current research, humans and the “uncanny” feeling accord an important role in the acceptance of technology. Social presence helps patients feel more heard by agents that personalize motivational messages to their situation, but human-like avatars, mismatched prosody, and latency can induce discomfort or erode trust, especially in emotionally vulnerable users. The clearest, focused on patient, design lesson is to calibrate realism (face, voice, dialog) to a comfort zone that sustains empathy without triggering the uncanny valley, often via stylized or “warm” designs and predictable turn-taking.

Voice interfaces can widen access for low-literacy users but introduce accent/noise errors and added latency; text interfaces aid auditability but can exclude users with visual or motor impairments. Hybrid, switchable modalities and local language models that learn an individual’s speech patterns are promising but not yet entirely implemented in healthcare. Similarly, hybrid architectures (systems implementing and synchronizing data from multiple streams) can mitigate outages and improve responsiveness, but introduce maintenance, standardization, and privacy trade-offs that must be handled explicitly within GDPR-style regimes and through explainable-AI safeguards.

From the patient’s vantage point, near-term contributions of VR/AI are:

- lowering the cognitive and logistic effort of self-management (automated tracking, structured skills practice),
- improving readiness and confidence through experiential learning (VR rehearsal, safe exposure),
- Offering timely, personalized micro-interventions (motivational prompts, coping scripts) that can soften daily symptom impact. These align with population-health goals even when disease-progression endpoints take years to detect.

The current state of studies suggests that personalization, empathic response, and adaptive difficulty are the levers that sustain engagement from the patient’s perspective. Evidence-based scripts and safety rules should be of the highest priority when it comes to using CAs and virtual coaches in healthcare settings.

The use of the technology described in this paper can therefore be concluded in the following principles:

1. Use chatbots/virtual agents to cover low-intensity support and coaching between the visits, with clear thresholds for human escalation and crisis protocols; prioritize modalities and devices that patients already own, reserve fully immersive VR for indications with strong engagement payoffs.
2. Prefer cloud designs to avoid therapy interruption; instrument VR sessions with maximum duration guidance (e.g., 15–20 min), live therapist view, and simple exit gestures. Track and mitigate cybersickness risk.
3. Keep avatar realism within a trust-promoting band; emphasize consistent prosody, turn-taking, and supportive nonverbal cues over lifelike appearance.
4. Personalize “what” (goals, prompts, examples), not “who” the agent is; avoid over-anthropomorphizing. Log and audit motivational messaging aligned with validated CBT/MI scripts.
5. Co-create with patients (and caregivers for pediatrics); prototype early to tune interaction burdens (voice vs. text), and publish technical details to enable replication.

## 7. Limitations

Much of the evaluated work is short-term, with modest samples and heterogeneous outcomes. Real-world effectiveness, particularly for multimorbidity, rural access, and long-horizon endpoints, remains not tested enough. Our synthesis emphasizes narrative integration; as such, it privileges cross-study themes (engagement, personalization, safety) while recognizing that condition-specific protocols will require tailored trials.

## 8. Conclusions

For patients living with chronic illness, VR and AI-driven agents can make the day-to-day work of self-management more doable, more timely, and more sustainable. The clearest gains today lie in engagement, symptom coping, and education; the highest risks lie in poorly calibrated social design, opaque data practices, and fragile infrastructure. Clinician adoption will hinge on healthcare workflows, training in ethical/technical limits, and the routine use of UX research and co-design to align systems with patient abilities and preferences. If these preconditions are met, the technologies reviewed here are well-positioned to meaningfully support prevention and quality of life across the chronic care continuum.

Future research should move beyond short-term outcomes and efficacy trials toward longitudinal evaluations in non-laboratory, clinical settings that examine sustained engagement, clinical outcomes (for example, reported lower levels of stress due to the use of VR), and unintended psychosocial effects over time. More specifically, randomized controlled trials and effectiveness–implementation studies are needed to assess how virtual agents perform as supportive “therapists” to standard care across different stages of chronic disease trajectories. Greater attention should also be given to comparative studies, contrasting text-based chatbots, embodied agents, and immersive VR systems to determine which design features meaningfully contribute to therapeutic alliance, adherence, and health-related quality of life. 

Besides the social perspective, the technical aspects should also be investigated, especially in the field of contextual interaction between the VR setting and LLM-based avatars. Choosing the proper software and headset for the virtual environment, adapting the proper LLM with the lowest rate of hallucination and highest number of tests in clinical settings, adjusting the design of the avatar that is supposed to communicate with the patients—these are all just examples that point out the complexity of implementing such technology in clinical settings.

## Data Availability

No new data were created or analyzed in this study. Data sharing is not applicable to this article.
